# Cardiac resident macrophages: key regulatory mediators in the aftermath of myocardial infarction

**DOI:** 10.3389/fimmu.2023.1207100

**Published:** 2023-06-30

**Authors:** Cong Chen, Jie Wang, Chao Liu, Jun Hu

**Affiliations:** Guang’anmen Hospital, China Academy of Chinese Medicine Sciences, Beijing, China

**Keywords:** cardiac resident macrophages, myocardial infarction, heart failure, pathophysiology, nanomedicine

## Abstract

Acute myocardial infarction (MI) is a prevalent and highly fatal global disease. Despite significant reduction in mortality rates with standard treatment regimens, the risk of heart failure (HF) remains high, necessitating innovative approaches to protect cardiac function and prevent HF progression. Cardiac resident macrophages (cMacs) have emerged as key regulators of the pathophysiology following MI. cMacs are a heterogeneous population composed of subsets with different lineage origins and gene expression profiles. Several critical aspects of post-MI pathophysiology have been shown to be regulated by cMacs, including recruitment of peripheral immune cells, clearance and replacement of damaged myocardial cells. Furthermore, cMacs play a crucial role in regulating cardiac fibrosis, risk of arrhythmia, energy metabolism, as well as vascular and lymphatic remodeling. Given the multifaceted roles of cMacs in post-MI pathophysiology, targeting cMacs represents a promising therapeutic strategy. Finally, we discuss novel treatment strategies, including using nanocarriers to deliver drugs to cMacs or using cell therapies to introduce exogenous protective cMacs into the heart.

## 1 Introduction

Acute MI is a critical and life-threatening condition caused by the rupture of vulnerable atherosclerotic plaques, leading to the formation of thrombus and subsequent obstruction of blood flow to the myocardium. This sudden reduction in blood flow can ultimately result in HF or death (1). Despite significant reductions in mortality rates through standard revascularization and pharmacological interventions, residual risks of HF remain elevated in patients with acute MI (2). Thus, there is a crucial need to explore innovative and effective approaches to preserve myocardial function and prevent the progression of HF in acute MI patients.

In 1968, Van Furth and Cohen first defined macrophages in mice as being derived entirely from mononuclear cells in the blood (3). In 1984, they discovered that macrophage populations in the spleen were unrelated to the influx of mononuclear cells in the blood, leading to the recognition of tissue-resident macrophages (4). Mammalian hearts contain a large number of cMacs. In the hearts of young adult mice, cMacs comprise 5-10% of non-myocardial cells and are composed of multiple distinct subpopulations with high heterogeneity and diverse lineages (5). Recent paradigm-shifting studies in mice have revealed that in addition to their immune function, cMacs have other protective roles in the cardiac injury process, including the clearance of necrotic myocardial cells and debris, promotion of vascular regeneration, maintenance of myocardial cell electrical stability, limitation of inflammation, and regulation of tissue remodeling (6). Therefore, effective elucidation of the biological information of cMacs in the context of MI will contribute to a better understanding of their protective effects in the pathophysiology of MI, and provide new avenues and methods for the diagnosis and treatment of MI.

## 2 Heterogeneity, origin, and spatial ecological niche of cMacs

### 2.1 Definition and genetic characteristics of cMacs

In the process of MI, the increase in the number of cMacs stimulated by myocardial injury or tissue remodeling is crucial for the progression and regression of tissue damage, although the relative heterogeneity and ontology of these cells are unknown. Environmentally dependent epigenetic mechanisms are among the foundations that regulate the transcriptional differences in cMacs heterogenous subpopulations (7). The traditional M1/M2 binary classification system for macrophages only applies to simplified environments *in vitro*, where macrophage heterogeneity is reduced, and the stimuli received by cells are controlled. However, in the complex *in vivo* environment during MI cardiac injury or tissue remodeling, the simplified M1/M2 definition cannot accurately describe the phenotypic characteristics of cMacs. Previous studies have classified human cardiac macrophages into three distinct subgroups (CCR2^+^ HLA-DR^low^;CCR2^+^ HLA-DR^high^;CCR2^−^ HLA-DR^high^) based on the markers C-C chemokine receptor type 2 (CCR2) and human leukocyte antigen-DR (HLA-DR), which is a human homolog of major histocompatibility complex class II (MHC-II) (8). In addition, the markers lymphocyte antigen 6 complex locus C (Ly6C) and MHC-II can be used to effectively distinguish four distinct subgroups of mouse macrophages (9).

The relevant study of differences between subpopulations of tissue macrophages aims to identify various subpopulations specific to different tissues, as well as unique markers used in each study. However, inconsistent methods among these studies lead to different markers being used and different subpopulations being identified, resulting in a lack of consistency (10, 11). Based on new genetic tools and unbiased single-cell RNA sequencing (scRNA-seq), Dick et al. studied cMacs transcriptional similarity across multiple mouse organs. They stratified cMacs into three subpopulations based on expression of phosphatidylserine receptor T cell immunoglobulin and mucin domain containing 4 (TIMD4), lymphatic vessel endothelial hyaluronan receptor 1 (LYVE1), folate receptor beta (FOLR2, TLF), CCR2, and MHC-II. These subpopulations included TLF^+^ cMacs (Markers:CX3CR1^lo^ TIMD4^+^ LYVE1^+^ TLF^+^ MHC-II^lo^ CCR2^-^), CCR2^+^ cMacs (Markers:CX3CR1^hi^ CCR2^+^ CD11c^+^ MHC-II^hi^ TIMD4^−^ LYVE1^−^ TLF^−^), and MHC-II^hi^ cMacs (CX3CR1^hi^ MHC-II^hi^ TIMD4^−^ LYVE1^−^ TLF^−^ CCR2^−^) (12). Among these three subpopulations, TLF^+^ cMacs are almost entirely maintained through *in situ* proliferation, while the self-renewal capacity of CCR2^+^ cMacs is limited and they are continuously replaced by monocytes (12). MHC-II^hi^ cMacs occupy a dynamic ecological niche that restricts the entry of monocytes to some extent, and therefore only some subpopulations are updated by monocytes (12). This study also suggests that CCR2^-/+^ is still a key marker for distinguishing between resident and non-resident macrophages and is considered conservative in humans, rats, and mice (13, 14). CCR2 may be involved in the classification of cMacs in later parts of this article (**Table 1**).

**Table 1:** Heterogeneity of cMacs.

Generalized macrophage subset	Macrophage subset	Surface markers	Proliferation	Origin	Cardiac Access Time
CCR2^-^ cMacs	TLF^+^ cMacs	CX3CR1^lo^ TIMD4^+^ LYVE1^+^ TLF^+^ MHC-II^lo^ CCR2^-^	Local proliferation	Yolk sac and fetal liver	E9.5-E10.5
	MHC-II^hi^ cMacs	CX3CR1^hi^ MHC-II^hi^ TIMD4^−^ LYVE1^−^ TLF^−^ CCR2^−^	20% to 40% are replenished by circulating monocytes		
CCR2^+^ cMacs	CCR2^+^ cMacs	CX3CR1^hi^ CCR2^+^ CD11c^+^ MHC-II^hi^ TIMD4^−^ LYVE1^−^ TLF^−^	Circulating monocytes	Fetal monocyte precursor cells	E14.5

cMacs, Cardiac resident macrophages; CD11c, Integrin alpha X; CCR2, CC- chemokine receptor 2; CX3CR, Chemokine (C-X3-C motif) receptor; LYVE1, lymphatic vessel endothelial hyaluronan receptor 1; MHC-II, major histocompatibility complex class II; TLF, folate receptor beta; TIMD4, T cell immunoglobulin and mucin domain containing 4.

### 2.2 Origin and maintenance of cMacs

Due to the fact that macrophages originate from at least three distinct lineages, the heterogeneity of cMacs may also be related to their origins and individual development (14–16). With the emergence of new technologies such as fate mapping and lineage tracing, we are now able to label and track the origin of cMacs, and monitor their phenotypic transitions during tissue development (17). The earliest macrophage precursor cells originate from the extra-embryonic yolk sac between embryonic day 7.0 (E7.0) and E9.0. With the development of embryonic blood vessels and the aorta-gonad-mesonephros (AGM) region, hematopoietic stem cells (HSC) are generated and settle in the fetal liver and eventually the bone marrow. Therefore, at least two mechanisms (yolk sac and fetal liver) can establish a resident population of macrophages before birth (18). The earliest CCR2^-^ cMacs originating from yolk sacs appear in the heart tissue at E9.5-E10.5, followed by CCR2^+^ cMacs from fetal monocyte precursor cells that take up residence in the heart at E14.5 (19).

TLF^+^ cMacs and MHC-II^hi^ cMacs can be classified into the range of CCR2^-^ cMacs, which originate from the yolk sac and fetal liver. CCR2^+^ cMacs are updated from monocyte precursors in circulation (12). Studies on lineage promotion using mouse models have implications for humans. Based on scRNA-seq, macrophage populations (most likely cMacs) can be detected in the human embryonic heart 5-7 weeks after conception, with cMacs possessing TLF^+^ gene features (similar to yolk sac macrophages) observed in the human fetal heart 7 weeks after conception (20–22). These subtle changes emphasize the need for multiple markers when identifying subpopulations, and their confirmation using high-dimensional transcriptomics. It is currently unknown whether cMacs from the yolk sac and fetal liver have functional differences when responding to inflammation or repair.

In addition, there is active competition among cMacs of different lineages. In humans and mice, CCR2^+^ cMacs are constantly replaced by circulating monocytes, even in CCR2^-^ cMacs, where MHC-II^hi^ cMacs are replaced by circulating monocytes at a rate of approximately 20% to 40% (12). However, the current issue is whether the observed cMacs turnover process during different developmental stages, as observed through gene fate mapping studies, is progressive or limited. For example, at a certain time point, a subpopulation of labeled macrophages may be replaced, but it is unclear whether this trend will continue or whether it was just a one-time event (23). Based on existing data, the turnover of CCR2^+^ cMacs is ongoing, while some CCR2^+^ cMacs undergo limited replacement. Only through separate analysis of these three cMacs subpopulations can their origins be accurately defined.

### 2.3 Ecological niche of cMacs

The functional heterogeneity of cMacs may also arise from different spatial ecological niches within the heart. The interaction between spatial ecological niches and cMacs within the heart may involve four aspects: (1) the ecological niche provides a physical attachment base or scaffold for cMacs; (2) the ecological niche provides nourishing factors for cMacs’ self-sustenance; (3) the ecological niche imprints cMacs’ tissue-specific identity through the expression of key transcription factors; and (4) cMacs, in turn, should provide benefits for their niche (24). Regarding the mechanisms underlying the trend of cMacs entering the niche, CCR2^-^ cMacs are established before birth and subsequently maintained independently of the supplement of blood monocytes during adulthood, whereas CCR2^+^ cMacs are driven to enter the heart by proteins such as CCR2.

**Figure 1:**
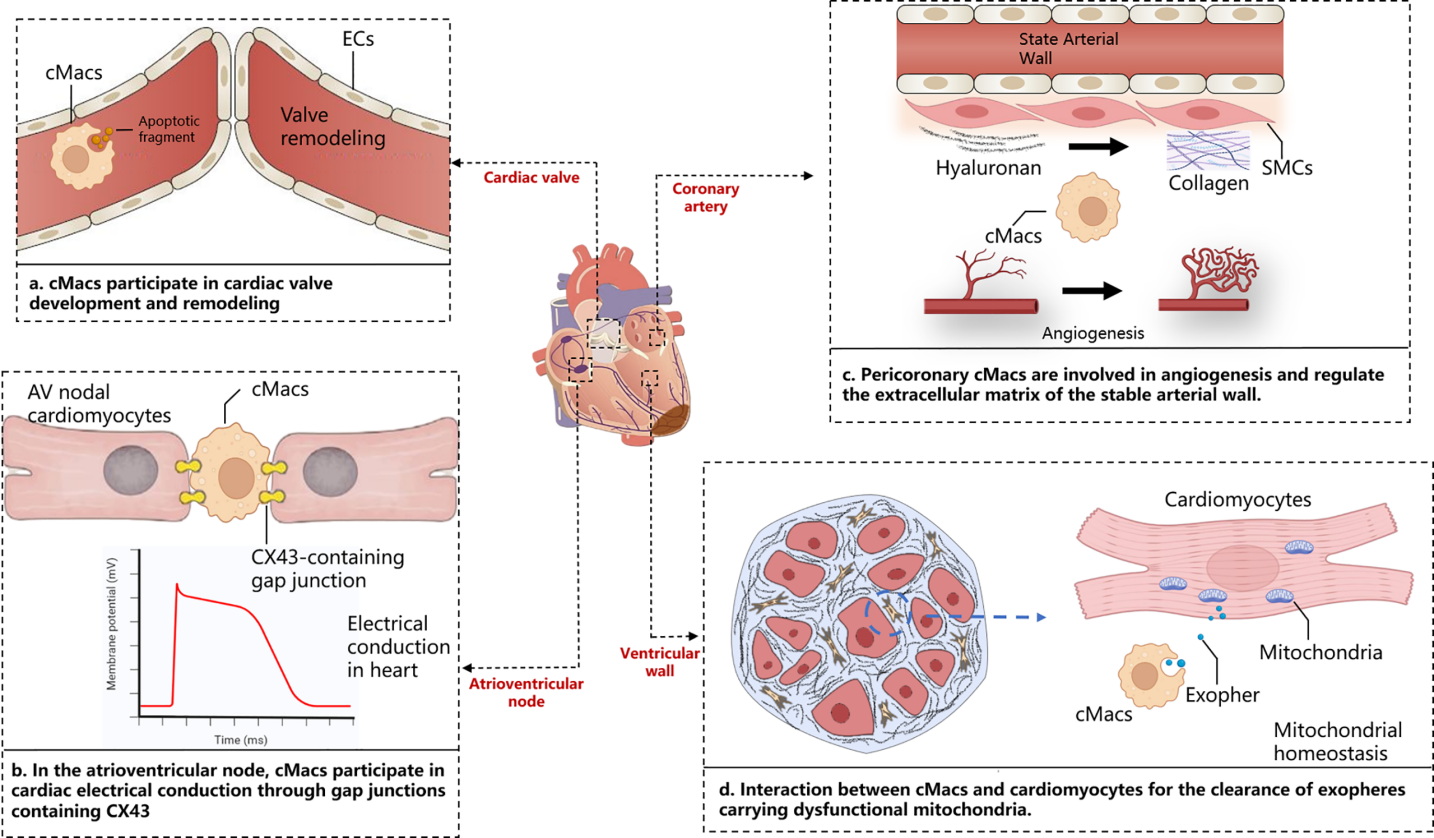
Cardiac niche with respect to cMacs. The figure depicts the macroscopic structure of the heart, including cardiac valves, atrioventricular node, ventricular wall, and coronary arteries, presented alongside magnified images of distinct tissue sections. These panels highlight the specific interactions between cardiac resident macrophages and cardiomyocytes in those areas. AV, Atrioventricular; cMacs, Cardiac resident macrophages; CX43, Connexin 43; ECs, Endothelial cells; SMCs, Smooth muscle cells.

Within the complex physical and pathological environment of the heart, cMacs exhibit distinct characteristics depending on their ecological niche. The heart is a highly vascularized organ, and a large number of CCR2- cMacs gather around blood vessels (19). These cMacs regulate vascular homeostasis by interacting with smooth muscle cells and collagen, as well as expressing lymphatic endothelial hyaluronan receptor-1 (LYVE-1) (25). cMacs also interact directly with lymphatic endothelial cells, regulating lymphatic growth and sprouting during embryonic heart development (26). cMacs in the heart valves play an important role in their development, repair, and injury response (27). During heart development, endocardial cushion cells undergo epithelial-to-mesenchymal transition (EMT) to generate mesenchymal cells, which then undergo a remodeling process to form mature, thin, and delicate heart valves. This remodeling process involves apoptosis, which cMacs may participate in by clearing apoptotic debris (28). In terms of cardiac conduction, CCR2- cMacs are highly enriched at the atrioventricular node, where they form gap junctions with conduction myocytes through connexin 43 (Cx43) and regulate their electrical activity through periodic depolarization (29). Lyve1^lo^ MHCII^hi^ cMacs(including CCR2^+^ cMacs and MHC-II^hi^ cMacs)have been reported to accumulate near nerve fibers in the tissue, but their specific functions in the heart under steady-state and disease conditions remain unclear (30). cMacs are present throughout all regions of the heart structure, and their ecological niche within the heart is a crucial determinant of their functional heterogeneity (**Figure 1**).

## 3 How cMacs regulate the pathophysiology of myocardial infarction

### 3.1 Inflammation response and recruitment of peripheral immune cells

Inflammation has harmful effects on injured myocardial tissue, but it is also essential for tissue repair (31). Different subgroups of cMacs may be a key mechanism to explain this paradox. Single-cell sequencing of cMacs in ischemic injury of the heart reveals differences in the fate of recruited monocytes between CCR2^-^ cMacs and CCR2^+^ cMacs (13). CCR2^+^ cMacs are important upstream mediators of the inflammatory response to myocardial injury (32). Damage-associated molecular patterns (DAMPs) released by dying myocardial cells activate Toll-like receptors (TLRs)/myeloid differentiation primary response 88 (MyD88)-dependent signaling in CCR2^+^ cMacs, leading to the release of cytokines and chemokines (13). CC chemokines (CCs) mediate the recruitment of a large number of pro-inflammatory CCR2^+^ monocytes and fill the ecological niche left by depleted CCR2^-^ cMacs after myocardial infarction, while CXC chemokines (CXCs) are associated with neutrophil infiltration (11). In contrast, CCR2^-^ cMacs have anti-inflammatory effects and can reduce the infiltration of CCR2^+^ monocytes. The molecular basis of the anti-inflammatory effect of CCR2^-^ cMacs may be related to the suppression of harmful type I interferons (IFNs) via the Nrf2 pathway dependent on transcription factor (33). However, CCR2^-^ cMacs rely on *in-situ* proliferation, and CCR2^+^ cMacs derived from recruited CCR2^+^ monocytes after myocardial infarction replace the original CCR2^-^ cMacs. Although some MHC-II^hi^ cMacs can be replenished through circulating monocytes, this cannot compensate for their loss in function. Is it due to epigenetic or post-transcriptional differences between pre-existing MHC-II^hi^ cMacs and recruited macrophages, or do they appear too late, only after additional tissue damage occurs?

**Figure 2:**
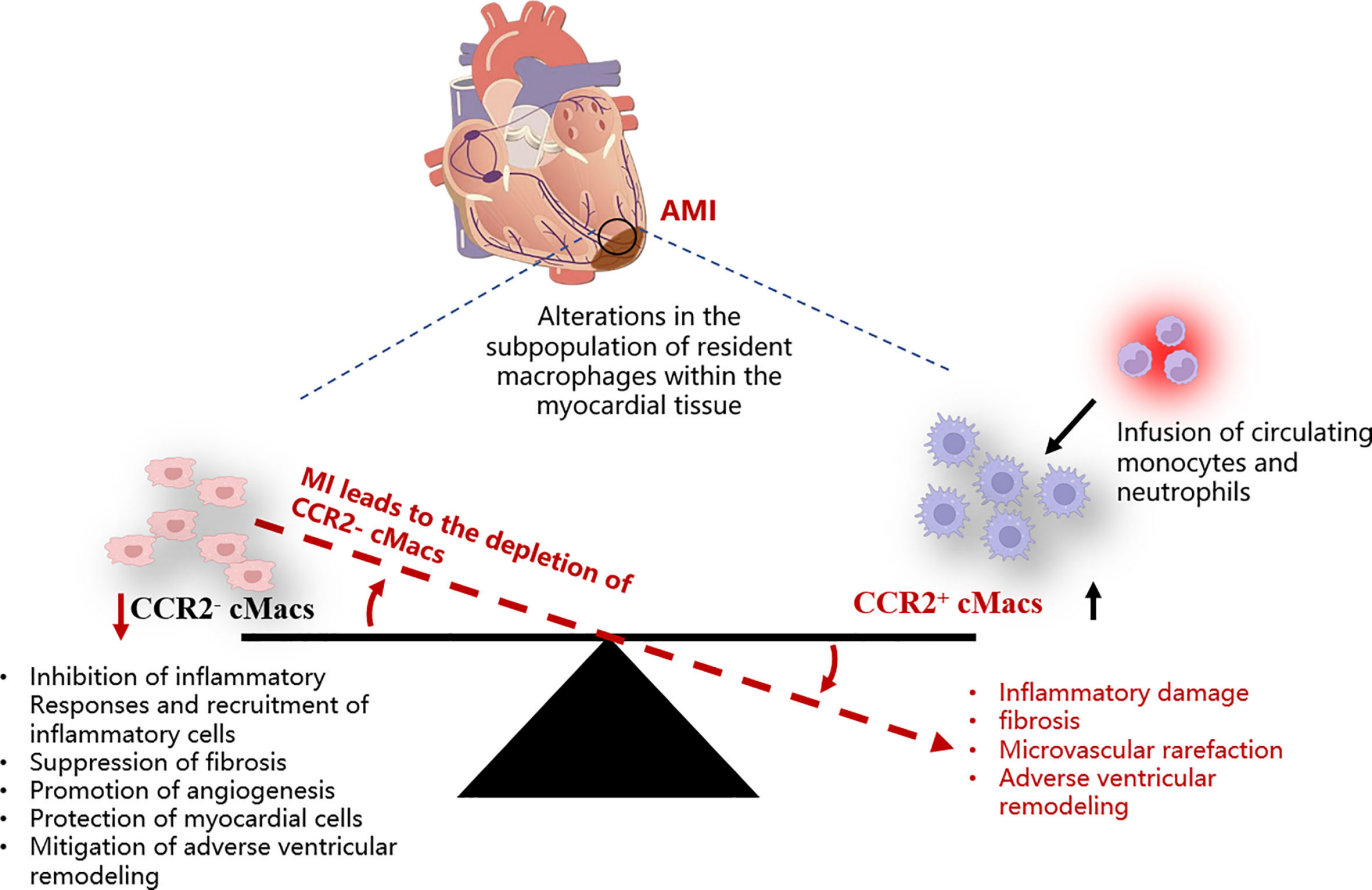
Schematic representation of the imbalance among different cMacs subpopulations within the myocardial tissue post-AMI. AMI, Acute myocardial infarction; cMacs, Cardiac resident macrophages; CCR2, C-C chemokine receptor type 2.

The quantity of CCR2^+^ cMacs present before MI is limited, and they may be an important early trigger of inflammation in ischemic conditions. CCR2^+^ cMacs are enriched in pathways associated with inflammatory body activation, pattern recognition receptor signaling, and cytokine production, as evidenced by transcriptional profiling (14, 34). Depletion of CCR2^+^ cMacs (based on Ccr2^DTR^ mice) before MI led to reduced monocyte recruitment and IFN-induced macrophage accumulation, resulting in improved cardiac function in an ischemic injury heart transplant model (donor-matching with the recipient) (13). This study compared the different effects of CCR2^-^ cMac depletion (CD169^DTR^) and CCR2^+^ cMac depletion (Ccr2^DTR^) on left ventricular function, myocardial remodeling, and monocyte recruitment. However, due to the lack of specificity in the methods used, the conclusion of this study was oversimplified: CCR2^+^ resident macrophages are also present in other organ tissues, and their depletion may affect the response to injury, not only limited to cMacs in the heart (32). Additionally, other cell types, including lymphocyte subsets, activated fibroblasts (FBs), and vascular cells, also express CCR2, and the CD169^DTR^ system may alter these cells, thereby affecting the conclusion (35, 36) (**Figure 2**).

### 3.2 Clearance of damaged cardiomyocytes and orchestration compensatory cardiomyocyte growth

During the acute inflammatory phase of MI, the number of necrotic and apoptotic cardiomyocytes is a key determinant of the severity of adverse ventricular remodeling and heart failure (37). Inefficient clearance of dying cardiomyocytes is also associated with suboptimal tissue remodeling after MI (38). Therefore, strategies that effectively clear dying cardiomyocytes may promote the resolution of inflammation and prevent extensive cell death, thereby slowing the progression of heart failure. Efferocytosis, or the engulfment of apoptotic cells or cellular debris, is a defining feature of macrophages and can sustain tissue homeostasis and trigger a cascade of responses in cardiomyocytes (39). Mer Tyrosine Kinase (MerTK), a surface receptor that recognizes “eat-me” signals, aids in the phagocytic clearance of apoptotic cells by MHC-II^lo^ CCR2^-^ cMacs (TLF^+^ cMacs) (40). MHC-II^lo^ CCR2^-^ cMacs possess the highest levels of MerTK and demonstrate stronger phagocytic activity in both steady-state and injury, as well as enhanced production of anti-inflammatory cytokines (40). However, clinical studies have shown that this beneficial phagocytic pathway is impaired after myocardial reperfusion, which occurs through the cleavage of the phagocytic receptor, MerTK. The loss of MerTK reduces the phagocytic activity of TLF^+^ cMacs in steady-state and biases the population towards MHC-IIhi cMacs, which are enriched with genes involved in antigen presentation, making the host more susceptible to infection with pathogens (14). The potential cause of MerTK cleavage in reperfusion injury is believed to be the recruitment of CCR2^+^ cMacs. This hypothesis is based on the proteolytic and proinflammatory phenotype of Ly6C^hi^ monocytes, which may cleave MerTK and affect the function of TLF^+^ cMacs (41). Another key protease that mediates cMacs efferocytic function is Legumain. Legumain has been identified as an essential lysosomal enzyme during cellular proliferation that is necessary to prevent the accumulation of apoptotic cardiomyocytes, prevent excessive leakage of apoptotic cell contents, and regulate proper resolution of inflammation (42). Lack of Legumain increases the recruitment and infiltration of CCR2^+^ cMacs, while downregulating the anti-inflammatory cytokine interleukin-10 (IL-10) and transforming growth factor-β (TGF-β), and upregulating proinflammatory mediators IL-1β, tumor necrosis factor-α (TNF-α), IL-6, and IFN-γ (42).

Terminal differentiated cardiac myocytes are unable to divide and must respond to disruptions in homeostasis through compensatory structural remodeling (instead of proliferation) in order to maintain cardiac function. Exhaustion of cMacs during MI exacerbates pathological growth of cardiac myocytes, indicating that cMac-driven cardiac myocyte growth is an example of compensatory hypertrophy (11). In the transverse aortic constriction (TAC) model, dual-tone protein (Areg) derived from Ly6C^lo^- macrophages is necessary for compensatory hypertrophy (43). Co-localization studies suggest that Ly6C^lo^-macrophages may be CCR2^-^ cMacs (44). Similarly, deletion of insulin-like growth factor-1 (IGF-1) from CCR2^-^ cMacs using the Cx3cr1^creERT2^ system demonstrates that IGF-1 derived from CCR2^-^ cMacs is necessary for adaptive cardiac myocyte growth and maintenance of cardiac function during AngII hypertension (despite the fact that most cardiac IGF-1 protein remains unchanged during disease) (45).

### 3.3 Cardiac fibrosis

Myocardial fibrosis is the expansion of the myocardial interstitium resulting from the net accumulation of extracellular matrix (ECM) proteins and is a critical pathophysiological factor that affects ventricular remodeling after MI (46). Based on histopathological analysis, cardiac fibrotic lesions can be classified into two distinct forms: “replacement fibrosis” and “interstitial fibrosis.” In MI, dead myocardial cells are replaced by collagen-based scars, resulting in reparative replacement fibrosis, which can maintain the structural integrity of the cavity and prevent catastrophic mechanical complications and is considered irreversible (46). The other form, interstitial fibrosis, mainly occurs in the distal area of the infarct zone and is the pathological basis for cardiac stiffness and poor ventricular remodeling, which is potentially reversible. Depletion of CCR2^-^ cMacs using CD169^DTR^ mice can induce myocardial fibrosis, indicating that CCR2^-^ cMacs may be its inhibitory factor (47). In other studies, depletion of CCR2^-^ cMacs using the Cx3cr1^creERT2^ and CD169^DTR^ systems results in the worsening of interstitial fibrosis between ventricular wall myocardial cells in hypertension and genetic cardiomyopathy, but this is limited to the vicinity of the infarct zone in MI (13, 45). This suggests that the anti-fibrotic function of CCR2^-^ cMacs may need to be activated under tissue injury conditions.

Continuous pressure and volume overload in MI lead to mechanical wall stress and inflammatory stimuli as the driving factors for activating cardiac FBs and inducing interstitial fibrosis (48). Confocal, two-photon and electron microscopy show sustained contact between cMacs and myocardial cells, suggesting a potential role in the communication between these two cell populations (49). CCR2^-^ cMacs interact with adjacent myocardial cells through adhesive plaque complexes and are activated in response to mechanical stretch via a TRPV4 (Transient Receptor Potential Vanilloid 4)-dependent pathway that controls the expression of growth factors (49). The mechanosensitive pathway also plays a key role in FBs activation (46). In terms of the inflammatory response, chemokines CC and CXC as well as recruited mononuclear and neutrophil cells are significantly correlated with myocardial fibrosis (50). As previously mentioned, CCR2- cMacs indirectly alleviate the pro-fibrotic effect of inflammation by inhibiting CCR2+ cMacs. In addition, beta-adrenergic stimulation can activate FBs and induce myocardial fibrosis in ventricular remodeling, which may be indirect through the release of fibrotic mediators from stimulated myocardial cells after myocardial cell necrosis or activation of immune cells (51, 52). In a study of mice lungs, fat, heart and skin, the authors induced ablation during the injury period using Lyve1^cre/GFP^ mice crossed with Slco2b1^flox/DTR^, and observed increased recruitment of neutrophil and inflammatory cells as well as increased fibrosis, demonstrating that cMacs expressing LYVE1 (TLF^+^ cMacs) can indirectly inhibit damage in β-adrenergic receptor-triggered cardiac fibrosis (30).

### 3.4 Risk of arrhythmia

**Figure 3:**
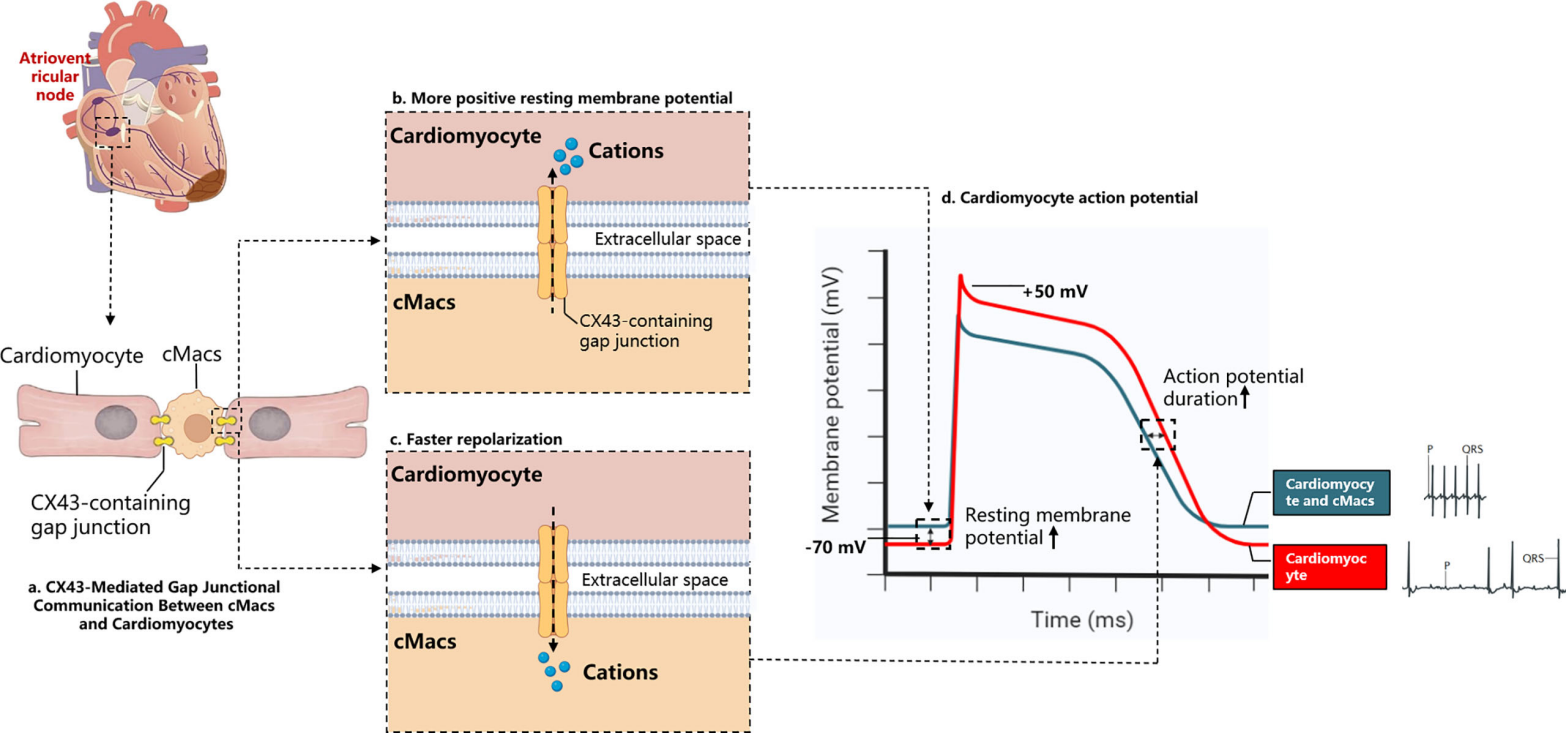
Electrochemical Interaction Between cMacs and Cardiomyocytes. **(A)** cMacs establish communication with cardiomyocytes via connexin 43 (CX43)-containing gap junctions, enabling the transfer of charges between cells; **(B)** In the resting membrane potential phase, macrophages possess a higher charge relative to cardiomyocytes’ resting membrane potential, leading to the migration of cations towards the cardiomyocytes, reducing their negative charge; **(C)** Upon cardiomyocyte depolarization, they become more positively charged than macrophages, instigating a transfer of cations back to the macrophages and supporting the rapid repolarization of the cardiomyocytes; **(D)** The electrocardiogram depicts a normal cardiac cycle (blue), contrasted with the ECG following macrophage depletion (red). The absence of cardiac macrophages prevents the propagation of the depolarization signal from atria to ventricles, resulting in an atrioventricular block. This condition, characterized by the decoupling of the atrial P waves and the infrequent ventricular QRS complexes. cMacs, Cardiac resident macrophages; CX43, Connexin 43.

When the heart experiences arrhythmias or conduction disorders, and the delivery of oxygenated blood stops, sudden cardiac death may occur (53). After MI, the uniformity of normal myocardial electrical activity is replaced by regional heterogeneity, which serves as the substrate for re-entry circuits (54). Various pathological changes may cause differences in the electrical activity of the myocardial region, which can contribute to the formation of re-entry circuits and ventricular arrhythmias. Examples of these changes include abnormal ion channel function and structural alterations in ion channels and gap junctions due to oxidation (55). Fibrosis of the myocardium and dying or dead cells may also slow down conduction, thereby promoting arrhythmic substrates (56). Nahrendorf and colleagues found that there are numerous CCR2- cMacs present in the distal atrioventricular node (AVN) of both mice and humans, and that these cMacs specifically express gap junction protein 43 (Cx43) at the point of contact with the myocardial cells (29). Through a series of *in vitro* models, Nahrendorf and colleagues demonstrated that cMacs communicate with coupled myocardial cells via electrical tension. cMacs connected to spontaneously beating myocardial cells via gap junctions containing Cx43 are synchronized in depolarization with coupled myocardial cells and have more negative resting membrane potentials than isolated macrophages (29). This research highlights the steady-state regulatory function directly executed by cMacs (**Figure 3**).

cMacs can prevent heart conduction block and ventricular arrhythmia through indirect functions. In the model of right heart pressure overload (pulmonary hypertension), macrophage-derived Areg has been shown to prevent fatal arrhythmias and sudden death (57). Areg is a key mediator that controls the phosphorylation and translocation of Cx43 in myocardial cells, and its deletion leads to the dissolution of gap junctions (57). Simon-Chica et al. revealed the mirror effect of plasma channel subunit expression, such as Kir2.1, Kv1.5, Kv1.3, Cx43, and Nav1.5, between myocardial cells and cMacs, which accelerates the repolarization of myocardial cells (58). Therefore, we can infer that cMacs that migrate to the injured site in MI and proliferate at the site can act as an electrical bridge in cardiac scar tissue, reducing the possibility of conduction block and arrhythmia by expressing Cx43 (59).

In addition, CCR2^+^ cMacs may also participate in the process of preventing arrhythmia in MI. Depletion of monocytes/macrophages before MI using CCR2^-/-^ mice led to ventricular arrhythmias induced by MI within 24 hours (60). However, under conditions of reduced blood flow and monocyte depletion, a decrease in cell debris clearance was observed as early as 5 hours after MI, suggesting that CCR2^+^ cMacs may participate in the clearance of necrotic debris. The specific mechanism is that CCR2^+^ cMacs may clear necrotic debris through phagocytic receptors CD36 and MerTK, thus preventing arrhythmia caused by MI (60). In future studies, the use of CCR2-based targeted systems may reveal the role of CCR2^+^ cMacs or circulating macrophages at an earlier time point.

### 3.5 Mitochondrial homeostasis

Cardiomyocytes are long-lived, terminally differentiated cells specialized for contraction. In addition to being primarily composed of myofilaments, these cells also possess a rich and complex mitochondrial network capable of generating sufficient energy to sustain cardiac output (61). Due to their terminal differentiation, cardiomyocytes have limited proliferative capacity and therefore cannot dilute accumulated waste within their subcellular compartments. Impaired mitochondrial function resulting from ischemic hypoxia and inflammatory injury following myocardial infarction is a key driver of cardiac dysfunction and ischemia-reperfusion injury (62). Recent studies have shown that cMacs actively engulf mitochondrial fragments released from cardiomyocytes to maintain muscle and stromal homeostasis (47). Using optical tissue clearance and genetic ablation to functionally characterize the cMac pool in steady-state, the authors observed a complex network of cMacs in close proximity to each cardiomyocyte. Depletion of cMacs through the CX3CR1-GFP knock-in mouse model and the CD169^DTR^ system led to progressive cardiomyopathy, decreased cardiac output, diastolic dysfunction, and hemodynamic compromise within three weeks (47). The key downstream effector of cMac uptake of mitochondrial fragments was the phagocytic receptor MerTK. Hearts with MerTK deficiency recapitulated several aspects of cMac depletion, including accumulation of damaged and energetically compromised mitochondria, metabolic dysfunction, activation of inflammasomes, and diastolic dysfunction (47). Among cMac subsets, CCR2^-^ cMacs exhibited a strikingly rich vesicular morphology, enriched in some genes associated with cell processing and demonstrating the highest efficiency in particle uptake *in vitro* (14).

### 3.6 Angiogenesis and extracellular matrix remodeling in blood vessels

Observations in animal models have revealed that after occlusion of the vasculature in MI, a large number of myocardial cells die in the infarct core, while new microvessels form in the infarct border zone, subendocardial interspace, and epicardium (63). Currently, the prevailing view is that the source of post-MI angiogenesis is sprouting from locally resident endothelial cells that survive ischemic injury (64). On the one hand, promoting angiogenesis can provide a pathway for inflammatory cells to enter and promote replacement of the necrotic area with granulation tissue, ultimately leading to the formation of a collagen-rich scar. On the other hand, it can facilitate gas exchange, nutrient diffusion, and waste clearance to meet the high metabolic demands of the inflamed site and limit the ongoing apoptosis or death of myocardial cells in the infarct border zone (48). cMacs located near blood vessels may play a critical role in cardiac angiogenesis in MI (31). Mechanistically, CCR2^-^ cMacs regulate coronary artery remodeling in a potentially blood flow-dependent manner, i.e., they promote dilation of well-perfused vessels preferentially (19). In the absence of CCR2^-^ cMacs, the proliferation rate of vessels in the lumen of the blood flow channel is slower, while vessels that are not perfused still proliferate rapidly, indicating that CCR2^-^ cMacs directly or indirectly regulate the proliferation of coronary artery endothelial cells. IGF1 and IGF2 may be potential mediators of CCR2^-^ cMacs-mediated coronary artery remodeling (19). In the heart overload pressure model after TAC, CCR2^-^ cMacs promote angiogenesis by expressing vascular endothelial growth factor A (VEGF-A), thereby increasing vascular density (65). In *in vitro* experiments, co-culturing CCR2^-^ cMacs with HUVECs (human umbilical vein endothelial cells) increases the meshwork area as well as the number of nodes, connections, and meshes to stimulate tube formation (65).

cMacs interact with vascular support cells to promote branching and growth of the perfused vascular system. The arterial blood flow, which is critical for delivering oxygen and nutrients to organs, is directly dependent on the tension and diameter of blood vessels. The extracellular matrix of arterial cells consists of elastic proteins and collagen fibers, which determine the elasticity or stiffness of blood vessels, thereby affecting the ability of arteries to dilate or constrict and regulate blood pressure and flow. A study demonstrated the role of CCR2^-^ cMacs expressing LYVE-1 in regulating the steady-state arterial matrix content and aortic diameter (25). CCR2^-^ cMacs regulate collagen protein expression in Smooth muscle cells (SMCs) through the binding of LYVE-1 to the hyaluronic acid (HA) pericellular matrix of SMCs and MMP-9-dependent protein hydrolysis (25). Previous studies have shown that macrophages regulate extracellular matrix composition in both steady-state and atherosclerotic arteries (66, 67). When smooth muscle cells and CCR2^-^ cMacs were co-cultured, they significantly reduced extracellular protein levels such as collagen and elastin, while increasing the expression and activity of MMP-9 (25).

### 3.7 Lymphangiogenesis and remodeling

The lymphatic system is critical for maintaining interstitial fluid homeostasis and fine-tuning immune responses by regulating the clearance of immune cells, cytokines, and antigens (68). The lymphatic network in the adult heart is dense in the ventricles and sparse in the atria (69). Remodeling of cardiac lymphatics occurs in many cardiovascular diseases characterized by edema and inflammation, including patients with ischemic heart disease or advanced heart failure (70). Therapeutic lymphangiogenesis in the heart has been proposed as a target for treating heart failure after myocardial infarction. Inducing therapeutic lymphatic vessel growth can accelerate the resolution of post-MI heart inflammation and reduce levels of T cells and pro-inflammatory macrophages in the infarcted left ventricle, thereby improving cardiac function (71). Therapeutic lymphangiogenesis in the heart requires sustained expression of VEGF-C, which is expressed by CD64^lo^ cMacs (likely mostly monocytes) that stimulate lymphatic vessel formation (72).

The myocardium is highly sensitive to lymphatic loss as an increase in cardiac water content by only 2.5% can result in a 30% to 40% decrease in cardiac output (73). Reactive lymphangiogenesis following MI prevents tissue edema and ensures proper myocardial contractility. However, this reactive lymphangiogenesis declines with the onset of heart failure (74). The abundance of lymphatic endothelial cells (ECs) and LYVE1^+^ cMacs is positively correlated following TAC, indicating that these cMacs may aid in lymphatic vessel formation (74). Culturing lymphatic ECs under conditions treated with LYVE1^+^ cMacs revealed that the key driving factors for lymphatic remodeling during hemodynamic stress were VEGF-C and fibroblast growth factor (FGF) secreted by cMacs (74). These findings demonstrate that cMacs indirectly coordinate lymphatic vessel formation during development and stress through the secretion of VEGFC acting on lymphatic ECs (**Table 2**).

**Table 2:** Regulatory mediators of cardiac macrophages in post-myocardial infarction pathophysiology.

Pathophysiology	Origin	Function	Key Mediators	Ref
Inflammation Response and Recruitment of Peripheral Immune Cells	CCR2^+^ cMacs	Induction of cytokine and chemokine release for recruitment of monocytes and neutrophils	MyD88	(13)
	CCR2^-^ cMacs	Anti-inflammatory effect	Nrf2	(33)
Clearance of Damaged Cardiomyocytes and Orchestration Compensatory Cardiomyocyte Growth	TLF^+^ cMacs	Mediating phagocytosis to clear cardiac cell debris	MerTK	(40)
	CCR2^-^ cMacs	Inhibit recruitment and infiltration of CCR2^+^ cardiac macrophages, promote anti-inflammatory mediators, and suppress pro-inflammatory mediators	Legumain	(42)
	CCR2^-^ cMacs	Facilitate compensatory structural remodeling of cardiomyocytes	Areg	(43) (44)
	CCR2^-^ cMacs	Promoting adaptive growth of myocardial cells during AngII-induced hypertension	IGF-1	(45)
Cardiac Fibrosis	CCR2^-^ cMacs	Detection of alterations in mechanical signaling in cardiomyocytes	TRPV4	(49)
Arrhythmia	CCR2^-^ cMacs	Electrically coupling cardiomyocytes with macrophages alters the electrophysiological properties of the coupled cardiomyocytes	Cx43	(29)
	CCR2^-^ cMacs	Regulation of phosphorylation and repositioning of Cx43 in cardiac myocytes	Areg	(57)
Mitochondrial Homeostasis	TLF+ cMacs	Identification and clearance of defective mitochondria transported by cardiac exophers	MerTK	(47)
Angiogenesis and Extracellular Matrix Remodeling in Blood Vessels	CCR2^-^ cMacs	Promoting angiogenesis and increasing vascular density	VEGF-A	(65)
	CCR2^-^ cMacs	Regulating collagen expression by binding to the pericellular matrix of smooth muscle cells with hyaluronic acid	LYVE-1	(25)
Lymphangiogenesis and Remodeling	CD64^lo^ cMacs	Stimulating lymphangiogenesis	VEGF-C	(72)
	cMacs	Adaptation of lymphatic remodeling	FGF	(74)

Areg, amphiregulin; CCR2, C-C chemokine receptor 2; CD64, Fc γ receptor I; Cx43, connexin 43; IGF-1, Insulin-like growth factor-1; MerTK, Myeloid-epithelial-reproductive tyrosine kinase; MyD88, myeloid differentiation primary response 88; Nrf2, Nuclear factor E2-related factor 2; TLF, folate receptor beta; TRPV4, Transient Receptor Potential Vanilloid 4; VEGF-A, Vascular endothelial growth factor A; cMacs, Cardiac Resident Macrophages.

## 4 Targeted therapeutic strategies for cMacs

Given the role that cMacs play in all pathological processes affecting the structure and/or function of the heart post-myocardial infarction, it is reasonable to suggest that strategies targeting cMacs could hold promising potential for advancing treatments for MI. Nevertheless, several unresolved issues remain: 1. While the myocardium provides a unique milieu, does there truly exist a uniquely cardiac-specific resident macrophage function?; 2. How can therapeutics be specifically targeted and delivered to distinct cMacs?; 3. What approaches can be utilized to modulate specific subsets of cMacs? Advanced targeting strategies, such as the binary Cre system, may offer improved specificity in delineating the role of cMacs within myocardial tissue, thereby enhancing our understanding and potentially informing the development of more effective therapeutic interventions (75). However, current research on targeted cMacs therapy has focused almost exclusively on interventions in CCR2^+^ cMacs. The subgroup of CCR2^-^ cMacs is actually the one that has a regulatory effect on post-MI myocardial tissue.

### 4.1 Nanoparticles

Nanomedicine has emerged as a highly promising tool for achieving targeted drug delivery by reducing toxicity. For example, nanocarriers selectively inhibit the transcription of the chemokine (C-C motif) ligand 2 (CCL2) in bone marrow niche endothelial cells, effectively suppressing the egress of monocytes and reducing their recruitment to the heart as macrophages (76). In another study, a siRNA carried by nanoparticles was shown to exhibit strong specificity for targeting macrophages, silencing the expression of Map4k4 and inhibiting the production of TNF-α and IL-1β (77). Flores et al. proposed a new therapeutic approach that targets macrophages with nanoparticles and disrupts the CD47-SIRPα signaling pathway, inducing macrophage apoptosis and clearing cells and debris in plaques to prevent their formation and progression (78). Getts et al. used immune-modifying particles (IMPs) to reduce the number of macrophages in cardiac tissue and alleviate inflammation (79). The results of this study showed that IMP treatment reduced the number of macrophages in cardiac tissue, alleviated cardiac inflammation, and improved cardiac function (79). In a mouse model of MI, the targeted delivery of siRNA against CCR2 using nanoparticles injected into ApoE^-/-^ mice was found to improve inflammation resolution, promote infarct healing, and slow the development of heart failure after MI (80).

### 4.2 Cell therapy

After myocardial ischemia reperfusion injury, injection of bone marrow mononuclear cells (MNCs) can promote recruitment of CX3CR1^+^ and CCR2^+^ macrophages in ischemic cardiac tissue (81). MNCs alter the function of cardiac FBs, leading to reduced deposition of fibrotic tissue and improved cardiac function (81). Cardiosphere-derived cells (CDCs) can regulate macrophage phenotype, thus exerting cardioprotective effects (82). Experimental evidence suggests that the use of CDCs in rat models of ischemia-reperfusion injury can reduce cardiomyocyte apoptosis and scar formation. Additionally, research has shown that if systemic macrophages are eliminated by administering clodronate, the benefits of CDCs will be completely lost (82).

## 5 Unresolved issues

Over the past 40 years, significant progress has been made in our understanding of the biology of cMacs and their impact on various physiological and pathological processes. However, there still exists a gap in our mechanistic understanding of cMacs function during fetal cardiac development and in the adult heart.

The use of Cre or DT (diphtheria toxin receptor (DTR) or diphtheria toxin (DT)) systems to control the expression of one or two genes in macrophages in mutant mouse models also has several drawbacks. Firstly, the depletion of macrophages can cause acute inflammation, severely affecting myocardial tissue (83, 84). Thus, it is almost impossible to separate this inflammatory process from the role of macrophages absent from myocardial tissue. Secondly, deleting a specific subset of macrophages intrinsically may lead to developmental defects and compensatory mechanisms in model animals, further masking the recognition of the steady-state functions of macrophages. Thirdly, in some DTR-based systems, circulating monocytes can supplement the missing cMacs in the system (85–87), and researchers need to repeatedly inject DT to maintain cMacs deletion in any DTR-based system, but mice may quickly develop immunity to DT.

In addition to the role of cMacs in myocardial infarction (MI), which is the main focus of this paper, it is worth noting that there may be potential implications for other forms of cardiac injury, which may also offer valuable insights into the roles of cMacs in cardiac injury and disease. For instance, the pathophysiological mechanisms underlying TAC or volume overload may differ from those of MI, but the involvement of cMacs in these processes might shed light on shared or distinct molecular and cellular pathways. These could be avenues for future investigation, which would not only expand our understanding of the multifaceted roles of cMacs in cardiac injury but also potentially offer new therapeutic targets. However, it should be noted that while our review may contribute to the broader understanding of cMacs in various forms of cardiac injury, the specific roles and mechanisms may differ depending on the type and severity of injury.

## 6 Conclusion

Our understanding of the role of cMacs in the injury, repair, and remodeling of the infarcted heart is constantly increasing, revealing new therapeutic targets for MI survivors. However, the pleiotropic effects of cMacs and their significant pathophysiological heterogeneity pose major challenges for the clinical application of cMacs-targeted therapies. Modulating cMacs may not only serve as a protective measure against remodeling and heart failure, but may also play a crucial role in achieving the grand goal of myocardial regeneration.

## Author contributions

The research project was designed by JW and CC; organized by JW and JH; reviewed and critiqued by CL. The first draft of the manuscript was written by JW, CC and JH, and reviewed and critiqued by CC. All authors contributed to the graphical analysis, drafting and critical revision of the paper, and agreed to take responsibility for all aspects of the work. All authors contributed to the article and approved the submitted version.
